# On the Medical Effects of Digitalis

**Published:** 1818-02

**Authors:** David Uwins


*For the London Medical and Physical Journal.*

### *On the Medical Effects of Digitalis*; by DAVID UWINS, M.D.

SOME months since the Editors did me the favour to print a communication of mine, on the subject of an Affection of the Head, which yielded while the patient was taking foxglove and mercury. In consequence, I suppose, of that paper, a gentleman in Sloane-street, whom I have not the pleasure of being acquainted with, sent a woman* to me about five weeks ago, stating to her that I had been successful in the

* Mrs. Ackland, No. 8, South-street, Chelsea, treatment of a case which was conceived to be water in the brain. The woman had a child in her arms, that had every appearance of being within a very little of death. It was two years old; had a depressed heavy countenance, limbs not larger than a child of two months, tumid hard abdomen, pulse running with a rapidity almost beyond counting, and, in short, as I have said, there was every appearance of rapid sinking. The mother stated to me, that she had lost an elder child, whose symptoms, as far as she could judge, were nearly the same as in the present case; and that she felt especially uneasy on the subject, since there was a difference of opinion among the medical gentlemen who saw that child on the actual nature of its disease,—one supposing it to be an affection of the head; the other asserting that the complaint existed in the stomach and bowels. The two questions, of course, which she was anxious for me to solve were, Do you believe the malady in the present case to be water in the head?—and do you think recovery at all probable? Upon examination of the child, and comparing its actual situation with the history of the complaint, I felt little hesitation in pronouncing the case not to be hydrocephalus, but tabes mesenterica. I ordered, in consequence, one drop of the College tincture of foxglove to be given three times a day, for about a week; and after that time two drops at each dose, should the child survive long enough for the experiment, of which I was exceedingly doubtful.

Since that time the mother has paid me two visits, with the infant in her arms, evidently in a state of progressive amendment. The pulse has become slower and more tonic, the limbs are less flabby, the abdomen more soft and equal, and the countenance much improved.*

Here I anticipate a smile from many of my readers—a smile of scepticism as to the influence of the digitalis in operating the alleged amelioration; and every reader has, of course, the liberty of drawing what inference he pleases from the recital of the circumstances: I only beg him to give me credit for the fidelity of my report; and shall now proceed to make good that engagement which met the Editors' approbation, of "putting together some further hints on the subject of medicinal operation upon the lymphatic system in young persons; on the connexion of tabes mesenterica with chronic hydrocephalus; and on the comparative virtues, as well as combined effects, of mercury, fox-

* The child has been brought to me this morning, in a state of decided amendment, and is now taking four drops of the tincture.

glove, and steel, in several disorders incident to children."*

An affection of the mesenteric glands, I need not observe, must, especially to a growing child, prove of necessity a prolific source of much and serious mischief, as it radically interferes with every process of nutrition and support, by preventing a due assimilation of food and proper supply of chyle to the blood. When we meet with this malady in infants, the indications of its treatment are also as obvious as the rationale of its essence. Nothing can be more plain than that the object of medicinal agency must be to restore tone and energy to the weakened lacteal glands. But the mischief is, that this want of energy does not always display itself in the shape of mere weakness: there is irritation, for the most part, as well as debility,—arterial action, as well as lymphatic torpor. In fact, the state of things is considerably analogous to that of dropsy; in which disease you often are called upon to contrive means for abating the excitation of the blood-vessels, and at the same time to bring the lymphatic organs into a condition of still greater excitation. Now, we know that both mercury and steel, under such circumstances, are often inadmissible, inasmuch as their irritating qualities are operative upon the nerves and arteries, as well as upon the absorbent system of vessels; and foxglove is frequently admissible and efficacious in dropsical states, apparently from the circumstance of different parts of the animal body possessing a different measure of susceptibility to its influence. I am aware that this notion has been ridiculed; but, in my mind, the ridicule has been unfounded and misapplied,—for, in fact, the substance in question is not the only agent which acts upon the system in the way now supposed. Let an individual, for example, be brought under the influence of fear,—and we often see that, while this feeling induces a state of comparative inaction in the blood-vessels, it at the same time excites in an extraordinary manner the absorbents. So it is with digitalis; and it was from the analogy just hinted at, between tabes mesenterica and dropsy, and from the conception that such, as just stated, is the relative operation of this medicine upon the latter disease, that I was first induced to make trial of it in the former; and the very first experiment I made of its virtues on this ground succeeded beyond my most sanguine anticipations. The youth, who was the subject of this trial, was in a condition very little better than the child whose case is the more immediate cause of the present

† See Medical and Physical Journal, vol. xxxvi. p. 103.

paper, and he is now the strongest and healthiest of the family. I began in this instance, as the child was older, with two drops of the tincture three times a day, and gradually increased it to ten or twelve at each dose.

Now, had I no other examples to adduce in favour of the foxglove than these, I am sensible that their testimony in its behalf would have but very little weight. The recovery of these children might be justly imputed to those accidental efforts of nature which often prove friendly to the physician, and gain a credit for medicine to which it is not legitimately entitled. But the fact is, that, although the cases alluded to have been deemed worthy of especial notice, in consequence of their very particular characters, they are not by any means the only ones from which I infer the efficacy of foxglove in mesenteric atrophy. Both in public and private practice, I am in the constant habit of its employment; and, although, as in all other instances of chronic disease, and *chronically* operating medicine, I am frequently disappointed in my hopes, I daily see reason to ratify my former assumption, that "I have witnessed its influence, *in small and gradually augmented doses*, with as much clearness and satisfaction as can be obtained in the instance of any medicine, whose operation is not immediately perceptible." The uncertainty of its action has been alleged against the employment of foxglove, and I recollect my friend Dr. Adams stating, in the Medical Society at Bolt-court, that this remarkable irregularity of effect constituted, in his mind, a sufficient condemnation of its use. To such authority I am ready to pay every respect; but I shall beg leave here to repeat what I then replied to Dr. A., that the accusation, although well-founded, considering the foxglove as an anti-inflammatory medicine, does not, in my judgment, apply to its exhibition in the gradual, stimulant, corrective manner in which I have suggested a trial of its virtues. That it is a medicine, the efficacy of which cannot be calculated on with any kind of certainty, I am ready to allow, and I have often been surprised at the eulogium passed upon it by the late Dr. Currie, who informs us, in his volumes on Cold Affusion in Fever, "that he has employed digitalis to a very considerable extent in inflammation of the brain, the heart, and the lungs; and has succeeded with it in situations where he should otherwise have despaired." It is, according to my observation, not by any means so applicable or so safe in decided and active inflammation as in those passive sub-inflammatory states which are attended with lymphatic disorder as their peculiar characteristics; and in these cases we ought to feel our way, as it were, by the gradual insinuation of the drug into the frame, for the purpose both of safely ascertaining whether or not it applies to the individual case under trial, and likewise for the purpose of insuring its stimulating efficacy.

I might lengthen this communication to the extent of a more detailed statement of those morbid conditions which seem rather to demand mercury and steel than the medicine more especially under notice. To do this, indeed, was my original design when I sat down to put these observations together. I find, however, that I must reserve such statement for another opportunity, and shall for the present conclude by making an observation or two on the connexion between hydrocephalus and tabes mesenterica. It appears to me that the latter often degenerates into the former, more, if I may so say, through the medium of the lymphatic system, than by the way of those digestive derangements which have been of late years so much dwelt on as the source of every thing that is amiss, both in the body and the mind. These derangements have from the first been talked and treated of in rather a vague manner; but, if I understand the import of the "digestive-organs" assumptions, it is this,—that derangements in the stomach occasion accumulations in the bowels, which give rise to sympathetic irritations in the nervous system, and these irritations form the whole secret and essence of all morbid phenomena. Now, in the present case, that of mesenteric atrophy, there is often nothing present to authorise the predication of any primary disease in the first passages: the disorder does not consist in faulty *digestion*, but in defective *assimilation*; there is a clean tongue, there is a good appetite, there is a due secretion of the gastric juice; but the *glandular* condition of the frame prevents the completion of the chyliferous process, or at least prevents the possibility of the chyle finding its way duly into the blood-vessels. Hence the disease, with all its consequences, either necessary and immediate, or remote and contingent; and hence the *modus operandi* of its termination in hydrocephalus, when that, as it not seldom does, happens to be the consequence of the original malady. It is, in this case, the lymphatic system which seems to take on the sympathetic irritation, and hydrocephalic effusions are the consequence, not of digestive derangements, but of glandular torpor. I ought not indeed to be too severe upon these ventricular notions, since I perceive they are a little losing their hold upon the minds of medical men, and that the blood-vessels are coming to be more fashionable as presumed sources of diseased states. May we not, however, now be generalising too much in this way? and may not the modern notions of vascular congestions and topical inflammations tend rather too much towards drawing our minds away from the proper contemplation of the peculiar susceptibilities of the nervous system on the one hand, and the lymphatic organization on the other?

1, *Thavies Inn*; *Jan.* 8, 1818.

